# Synaptic Elements for GABAergic Feed-Forward Signaling between HII Horizontal Cells and Blue Cone Bipolar Cells Are Enriched beneath Primate S-Cones

**DOI:** 10.1371/journal.pone.0088963

**Published:** 2014-02-20

**Authors:** Christian Puller, Silke Haverkamp, Maureen Neitz, Jay Neitz

**Affiliations:** 1 Department of Ophthalmology, University of Washington, Seattle, Washington, United States of America; 2 Neuroanatomy, Max Planck Institute for Brain Research, Frankfurt am Main, Germany; University Zürich, Switzerland

## Abstract

The functional roles and synaptic features of horizontal cells in the mammalian retina are still controversial. Evidence exists for feedback signaling from horizontal cells to cones and feed-forward signaling from horizontal cells to bipolar cells, but the details of the latter remain elusive. Here, immunohistochemistry and confocal microscopy were used to analyze the expression patterns of the SNARE protein syntaxin-4, the GABA receptor subunits α1 and ρ, and the cation-chloride cotransporters NKCC and KCC2 in the outer plexiform layer of primate retina. In macaque retina, as observed previously in other species, syntaxin-4 was expressed on dendrites and axon terminals of horizontal cells at cone pedicles and rod spherules. At cones, syntaxin-4 appeared densely clustered in two bands, at horizontal cell dendritic tips and at the level of desmosome-like junctions. Interestingly, in the lower band where horizontal cells may synapse directly onto bipolar cells, syntaxin-4 was highly enriched beneath short-wavelength sensitive (S) cones and colocalized with calbindin, a marker for HII horizontal cells. The enrichment at S-cones was not observed in either mouse or ground squirrel. Furthermore, high amounts of both GABA receptor and cation-chloride cotransporter subunits were found beneath primate S-cones. Finally, while syntaxin-4 was expressed by both HI and HII horizontal cell types, the intense clustering and colocalization with calbindin at S-cones indicated an enhanced expression in HII cells. Taken together, GABA receptors beneath cone pedicles, chloride transporters, and syntaxin-4 are putative constituents of a synaptic set of proteins which would be required for a GABA-mediated feed-forward pathway via horizontal cells carrying signals directly from cones to bipolar cells.

## Introduction

Horizontal cells receive glutamatergic input from photoreceptors and in return provide inhibition laterally through the outer plexiform layer (OPL). This feedback occurs at their dendrites, which form contacts with cones, or at the axon terminals of axon-bearing horizontal cell types, which are connected to rods. Lateral horizontal cell feedback at the level of the photoreceptors [1]–[4] forms the basis of center-surround receptive field organization in retinal cells. Horizontal cells mediate surround suppression and cone opponency depending on stimulus conditions, which is then inherited from the cones to bipolar cells [5]–[8] and finally to ganglion cells (e.g. [9]–[12]). The mechanism by which horizontal cells perform this task is still in debate. Originally, these cells were thought to release the inhibitory neurotransmitter gamma-aminobutyric acid (GABA) onto cones, an assumption supported by reports showing GABA-mediated currents in isolated turtle and cultured porcine cones [13], [14]. Mammalian horizontal cells, including those of primate retina, have been shown to express the GABA-synthesizing enzyme glutamic acid decarboxylase (GAD) and accordingly they were found immunopositive for GABA and the vesicular GABA-transporter vGAT (e.g.[15]–[27]). However, the proposed GABAergic feedback from horizontal cells onto cones did not withstand further functional investigation in primate and other species [2], [3], [28]. The GABA hypothesis has been replaced by two other, not necessarily mutually exclusive, proposals for horizontal cell feedback mechanisms: one involving ephaptic feedback via connexin hemichannels [29] and the other involving pH changes in the synaptic cleft [30]. Both mechanisms finally influence the cone calcium currents and thereby the glutamate release from cones. Thus, GABA has largely been ruled out as the mediator of horizontal cell feedback onto cones. The idea of GABAergic signaling in the outer retina has fallen out of favor, and the reason for the presence of GABA synthesis and the corresponding release machinery in the outer retina remains a mystery.

There is, however, the possibility that GABA release by horizontal cells plays an alternate role, in which the target is not the cones, but the bipolar cells. Ultrastructural studies in cat and rabbit retinas revealed synaptic contacts between horizontal and bipolar cells in the proximal OPL [31]–[33]. Postsynaptic densities were found in dendrites of bipolar cells, which were opposed to vesicle clusters at the presynaptic site. The presynaptic partners were then identified as horizontal cell processes. The formation of synapses by horizontal cells at this level of the OPL is not surprising. It is well known that the horizontal cell synaptic architecture is highly complex, and synaptic specializations away from their contacts with photoreceptors have been described in human and non-human primates [34], [35]. At the level of the desmosome-like junctions approximately 1.5 µm beneath the cone pedicle base, horizontal cells also form glutamate receptor bearing postsynapses in close proximity of electrical synapses [20], [36]. Further, light and electron microscopic studies of primate retina have shown that GABA receptors are expressed by ON and OFF bipolar cells at these sites beneath the cone pedicle base [20], [37]. Different kinds of chloride transporters are expressed in dendrites of the two types of bipolar cells [38] in order to maintain proper center-surround opponent interactions via the feed forward pathway, i.e., in response to light offset in the surround, OFF bipolars hyperpolarize and ON bipolar cells depolarize. The potassium-chloride cotransporter KCC2 is located on OFF bipolar cell dendrites and extrudes chloride from the dendritic shaft, so that GABA release would elicit the typical inhibitory effect. ON bipolar cells, however, express the cation-chloride cotransporter NKCC, leading to an accumulation of chloride within the dendrites so that the chloride reversal potential is shifted to values positive to the resting potential [39]. Accordingly, GABA then elicits the efflux of chloride and an excitatory effect in the postsynaptic cell. Functional evidence for GABA-mediated excitation in rod and ON cone bipolar cell dendrites has been shown in studies of adult mouse retina [40]–[42], and it is also known from other regions of the (adult) central nervous system that GABA can cause neurons to depolarize [43]. In summary, horizontal cells likely provide feedback inhibition directly to cones in a non-GABAergic pathway, and in a separate GABAergic pathway they provide direct feed-forward excitation or inhibition to bipolar cells, depending on the bipolar cell type (ON vs. OFF).

Retinas of most mammalian species possess two types of horizontal cells, axonless A- and axon-bearing B-type cells, which correspond to primate HII and HI horizontal cells, respectively (reviewed in [44]). Despite this formal analogy, primate horizontal cells are unique among mammals, considering their specialized connectivity pattern. HI cell dendrites form contacts with L- and M-cones but largely avoid S-cones while the axon terminal is connected to rods. HII cells, however, preferentially contact S-cones with their dendrites and axon-like processes, but receive additional input from L- and M-cones [45]–[52]. The horizontal cells of many mammals do not exhibit any kind of cone selectivity. However, horse and ground squirrel retinas possess a ‘true blue’ horizontal cell type, which is solely connected to S-cones [53]–[55]. The complex pattern of cone-type selective horizontal cell circuitry is reflected in one key feature of the primate retina, that is, processing of spectrally opponent light signals. Evidence exists that HI cells are responsible for M:L cone opponency in the midget pathway [56]. However, the role of HII cells in the S-cone pathway is unknown. Therefore, the primate retina is of special interest with regard to the role of horizontal cells and the underlying synaptic architecture.

Here, we have used immunohistochemistry on retinas of adult macaque monkeys to investigate if synaptic components putatively involved in GABAergic signaling exist in the primate OPL. We have focused on syntaxin-4, which has been hypothesized to play a role in vesicular GABA release by horizontal cells [15], [57]. Syntaxins are well known for their role as soluble NSF-attachment protein receptor (SNARE) core complex proteins in vesicular trafficking and exocytosis (reviewed in [58]). Four syntaxin isoforms are expressed with only little overlap by different neuronal cell types in mouse retina [59]. In the OPL of different mammals including mouse and rabbit, syntaxin-4 has been shown to be expressed by horizontal cells [15], [57], [59] but its functional role in horizontal cells and its distribution in primate retina remain unknown. We further investigated the localization of postsynaptic elements required for a GABAergic feed-forward pathway from horizontal to bipolar cells, including GABA receptors and the cation-chloride cotransporters NKCC and KCC2.

## Materials and Methods

### Animals and Tissue Preparation

Retinas of adult macaque monkeys (*Macaca fascicularis, nemestrina,* and *mulatta*) were obtained through the Tissue Distribution Program of the National Primate Research Center at the University of Washington (WaNPRC). Animals are housed in pairs, and may additionally be in grooming contact with other animals. Animals are fed monkey chow and a variety of food treats including fresh fruits, vegetables and nuts. The WaNPRC employs environmental enhancement staff who provide animals with a variety of perches, toys, foraging toys, and food treats on a daily basis. Animals are monitored daily by Primate Center staff for health and well-being. Ketoprofen or other appropriate pharmaceuticals are administered to animals at the discretion of the WaNPRC veterinary staff to alleviate pain. The tissue was harvested from animals that were involved in other research and have reached a study endpoint of euthanasia. These prior experiments were not related to the present study and were individually approved by the University of Washington Institutional Animal Care and Use Committee. In other cases, veterinary staff has determined that euthanasia is required due to disease, which did not occur as part of the present study.

The eyes were enucleated, the retinas immediately dissected, and after initial perfusion with carbogenated, bicarbonate-based Ames medium, the tissue was then transferred to the fixative (see below). Animals from which eyes were obtained were euthanized by an overdose of sodium pentobarbital. All procedures were in accordance with guidelines of the University of Washington Institutional Animal Care and Use Committee.

Clomeleon-1 (Clm1) mice were deeply anesthetized with isoflurane and decapitated before tissue dissection. Clm is a genetically encoded ratiometric fluorescent indicator for chloride under control of the thy1 promoter [60]. In these animals, S-cone selective type 9 bipolar cells express Clm, and the GFP-antibody enhanced staining of their dendrites was used to locate S-cone pedicles [54], [61]. All procedures were carried out in accordance with institutional guidelines of the Max-Planck-Institute for Brain Research, Frankfurt, following the standards described by the German animal protection law (*Tierschutzgesetz*). Euthanasia of mice for harvesting retinas used in this study has been approved by the animal welfare officer of the Max-Planck-Institute for Brain Research and reported to the local authorities (*Regierungspraesidium Darmstadt*).

Eyes of adult 13-lined ground squirrels (*Spermophilus tridecemlineatus*) were obtained from the captive breeding colony at the University of Wisconsin, OshkoshBiology. The animals were decapitated, the eyes enucleated, the anterior segments removed, and the posterior eyecups immersion-fixed (see below). Procedures were approved by the Institutional Animal Care and Use Committee at the University of Wisconsin, Oshkosh.

After harvesting the tissue as described above, the posterior eyecups (mouse and squirrel) or naked retinas (monkey) were immersion fixed in 4% paraformaldehyde in 0.1 M phosphate buffer (PB), pH 7.4, for 10, 15, or 30 minutes at room temperature (RT). Following fixation and washing in PB, retinas were dissected from the eyecup if necessary, cryoprotected in graded sucrose solutions (10, 20, 30% w/v), and stored in 30% sucrose at −20°C until use. Retinal pieces were either used as a wholemount or sectioned vertically at 16 µm or horizontally at 30 µm using a cryostat (Leica Microsystems).

### Antibody Characterization

The primary antibodies used in the present study are listed in Table 1. All these antibodies have been characterized in other studies of mammalian retina and the staining patterns observed here were consistent with previous reports.

**Table 1:** Primary antibodies.

Antibody	Species, type, workingdilution	Immunogen	Source, catalog number
CaBP	Mouse, clone CL-300, 1∶1000	Calbindin-D-28K from chicken gut	Sigma, C8666
GABA_A_-R α1	Mouse, clone bd24, 1∶100	Purified GABA/benzodiazepine receptor frombovine cortex	Millipore, MAB339
GABA_C_-R ρ1	Rabbit, polyclonal, 1∶100	N-terminus (16–171) of recombinant rat GABA_C_-R ρ1	Ralf Enz, Friedrich-Alexander-University, Erlangen-Nuremberg, Erlangen, Germany
GFP	Goat, polyclonal, 1∶1000	Green fluorescent protein from *Aequorea victoria*	Rockland, 600-101-215
GluR3	Goat, polyclonal, 1∶300	C-terminal peptide of human GluR3(TNTQNYATYREGYNVYGTE)	Santa Cruz, sc-7612
KCC2	Rabbit, polyclonal, 1∶200	His-tag fusion protein corresponding to aa 932–1043of rat KCC2	Millipore, 07–432
NKCC	Mouse, monoclonal, 1∶100	38 kDa C-terminal fragment of human NKCC1cotransporter	Developmental Studies Hybridoma Bank, T4
Parvalbumin	Mouse, monoclonal, 1∶10,000	Carp muscle parvalbumin	Swant, 235
S-opsin	Goat, polyclonal, 1∶100	N-terminal peptide of human blue-sensitive opsin(EFYLFKNISSVGPWDGPQYH)	Santa Cruz, sc-14363
Syntaxin-4	Rabbit, polyclonal, 1∶500	Corresponding to aa 2–23 of rat or mouse Syntaxin-4	Millipore, AB5330

Specificity of the syntaxin-4 antibody has been shown by Western blots of mouse retina and brain, where it recognized a single band at the appropriate molecular weight [59]. Immunostaining with syntaxin-4 shows the same staining pattern in retinas of guinea pig [15] mouse, rat, rabbit [57], [59] macaque, and ground squirrel (this study). Preadsorption of the antibody with the corresponding control peptide blocked all staining in rabbit retina [57].

Specificity of GABA_A_-R α1 (clone bd24) antibodies has been tested in Western blots of human and bovine brain where it recognized a single band at 50 kDa [62], [63]. This antibody has been applied to macaque retina before [20] and yielded the same staining as shown in our study.

Antibodies against GABA_C_-R ρ1 subunits have been produced and characterized by Enz and colleagues [64], where it has been shown to detect ρ1 at 50 kDa, in addition to ρ subunits 2, and 3, but not other GABA_A_ or glycine receptor subunits on Western blots with human embryonic kidney cells, transfected with cDNA of the corresponding proteins. There, further experiments revealed similar immunostaining patterns in rat, rabbit, and monkey retinas. Specificity tests were also performed in Western blots using retinal lysate from rat and degu [65]. Immunohistochemical results of the aforementioned studies and of [20] match the data presented herein.

Common GFP antibodies were applied to enhance the fluorescence signal in retinas of Clm1 mice [61], [66], [67].

Antibodies against GluR3 were used to identify the plexiform layers and their borders. Labeling of mouse retinas with GluR3 revealed the expected synaptic stain, and preadsorption controls with the immunizing antigen eliminated staining [68]. The same synaptic staining was observed in macaque retina, and Western blots with GluR3 showed a single band at the predicted size between 100 and 110 kDa [69].

The NKCC (T4) antibodies used in this study are directed against both NKCC isoforms (1 and 2) [70] and have been shown to produce specific staining of a band above 140 kDa in Western blots of mouse, rat, rabbit, and ferret retinas and brain [38], [71]. NKCC staining has been shown before in macaque retina [38] and the overall staining pattern presented in our study closely resembles immunohistochemical data from that earlier study. However, immunostaining in mouse retina yielded controversial results regarding the specificity of this antibody [72], [73]. Nevertheless, functional experiments unambiguously showed the expected effect of NKCC in mouse ON bipolar cell dendrites [40], [42] and the NKCC-T4 antibodies were raised against primate NKCC. Thus, we conclude that despite the above mentioned discrepancy in mouse, the anatomical data presented by Vardi and colleagues [38] and herein are valid.

Specificity of antibodies against the potassium-chloride cotransporter KCC2 has been tested in Western blots of whole brain and retina lysates of various mammals where it recognized a single band at 140 kDa [38], [74]. Previous immunohistochemical staining of macaque retina with KCC2 [38] yielded results similar to what is shown herein.

Antibodies against S-opsin were used to label the corresponding cones including their pedicles in macaque and ground squirrel [69], [75], [76]. The specificity of the antibody was tested by the manufacturer with a Western blot analysis using mouse retinal extract, revealing a single band at the predicted size of 40 kDa.

Antibodies against the calcium-binding proteins calbindin (CaBP) and parvalbumin are well-established as horizontal cell markers in primate retina. CaBP labels HII horizontal cells (besides cone photoreceptors and two bipolar cell types), while parvalbumin labels both HI and HII horizontal cells [45], [77], [78]. The particular calbindin and parvalbumin antibodies which we have used here specifically stain the ^45^Ca-binding spot of calbindin-D (MW = 28,000, pI = 4.8) or of parvalbumin (MW 12,000 and IEF 4.9), respectively, in a two-dimensional immunoblot according to the information provided by the manufacturers.

### Immunohistochemistry and Confocal Microscopy

Immunocytochemical labeling was performed using the indirect fluorescence method. Sections were incubated overnight at RT with primary antibodies in 5% normal donkey serum (NDS), 1% bovine serum albumin (BSA), and 0.5–1% Triton X-100 in PB. After washing in PB, secondary donkey antibodies conjugated to Alexa TM 488, 568, or 594 (Invitrogen) were applied for 1 hour RT at a dilution of 1∶500. Retinal wholemounts were incubated freely floating for 2 days RT or for up to 5 days at 4°C in a mixture of primary antibodies containing 5% NDS, 1% BSA and 1% Triton X-100, followed by a 3-hour incubation in a mixture of secondary antibodies at RT.

Images were taken using an Olympus FV1000 confocal microscope equipped with an argon and a HeNe laser. High-resolution scanning of image stacks was performed using an Olympus UPlanSApo 60x/1.35 oil immersion objective with at least 1024×1024 pixels and a z-axis increment of 0.25 µm. When maximum intensity projections of image stacks are shown, 2–4 serial optical sections were collapsed into a single plane. Brightness and contrast of the final images were adjusted using Adobe Photoshop.

## Results

### Syntaxin-4 in Primate Retina

**Figure 1:**
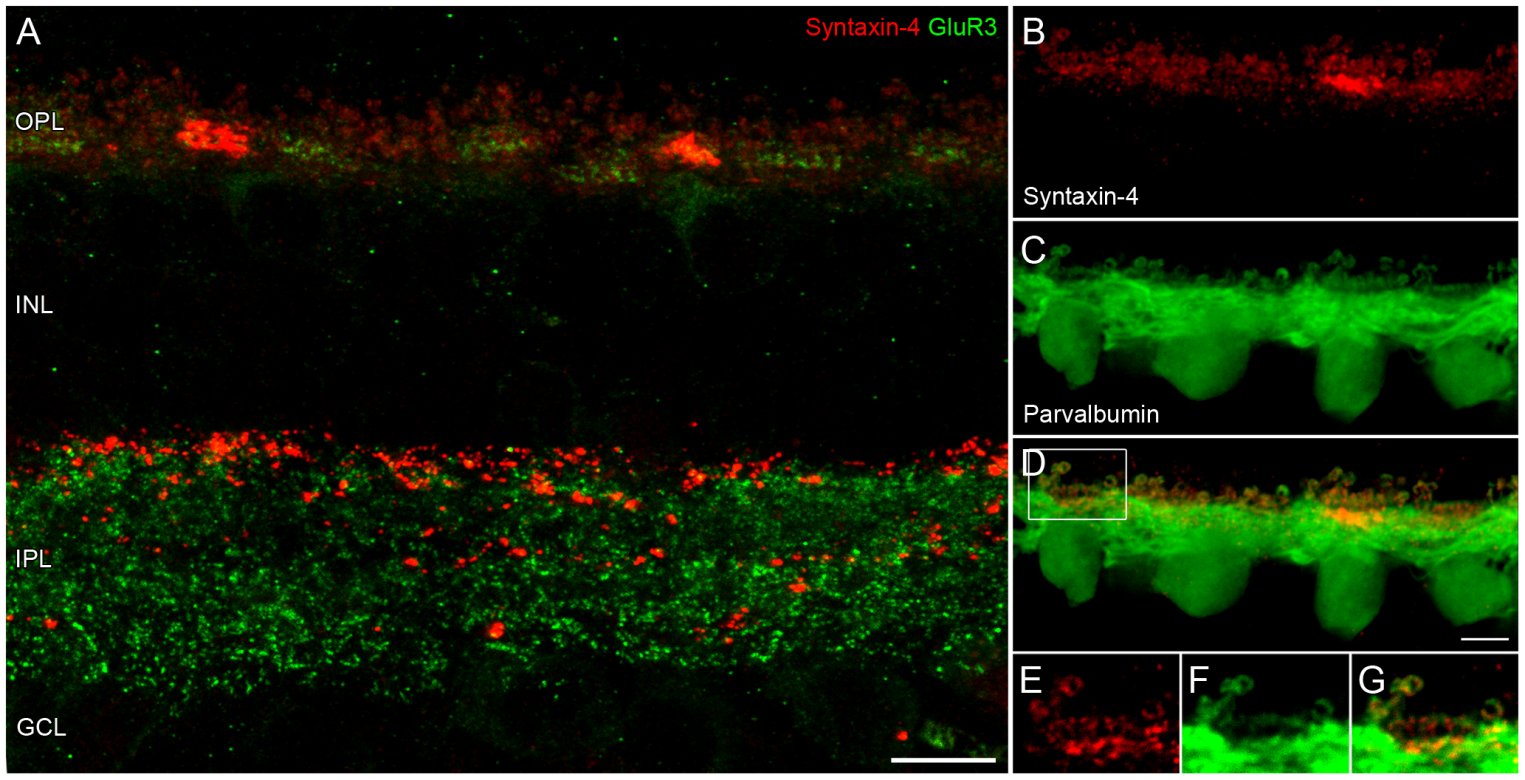
Syntaxin-4 in primate retina. **A:** Overview of syntaxin-4 staining (red) in a maximum intensity projection of an image stack from vertical cryostat sections of macaque retina. Labeling of GluR3 (green) indicates the inner and outer plexiform layers (IPL/OPL) and their margins. INL: inner nuclear layer, GCL: ganglion cell layer. **B–G:** Double labeling of syntaxin-4 and horizontal cells with antibodies against parvalbumin. Syntaxin-4 is colocalized with horizontal cell dendrites and axon terminals. **E–G:** Magnification of a single optical slice from the boxed region in D. Scale bars: 10 µm in A, 5 µm in D.

In contrast to other mammalian species, to date the distribution of the SNARE protein syntaxin-4 remains unknown in primate retina. To initially identify the overall expression pattern of syntaxin-4, vertical sections of macaque retina were immunolabeled against this protein and counterstained with GluR3 in order to label the synaptic layers (Fig. 1A). In the OPL, large, brightly labeled aggregates were surrounded by a weaker but dense and often punctate staining. In the inner plexiform layer (IPL) syntaxin-4 labeling appeared as mostly large puncta, scattered throughout the full depth of the IPL. The majority of puncta were clustered at the distal border towards the inner nuclear layer. Double labeling of syntaxin-4 and tyrosine hydroxylase, a marker for dopaminergic amacrine cells, revealed a close association of syntaxin-4 with this cell type rather than a colocalization in mouse IPL [59]. We repeated this experiment in macaque with the same outcome (data not shown). Next, double labeling of syntaxin-4 and the calcium-binding protein parvalbumin, a marker for horizontal cells [77], was performed to determine if the syntaxin-4 immunoreactivity in the primate OPL arises from its expression by these cells. Figure 1B–D shows the expected overlap of syntaxin-4 and parvalbumin. Syntaxin-4 labeling was clustered at the hooked axon-terminal endings of horizontal cells postsynaptic to rods, and in two bands at cone pedicles (Fig. 1E–G). The upper band coincided with the dendritic tips of horizontal cells, while the lower band was located at the approximate level of their desmosome-like junctions. This double-banded appearance of horizontal cell synaptic proteins at cone pedicles is well-known from the spatial organization of their glutamate receptors [20], [69]. Their expression is spatially restricted to the dendritic tips and the desmosome-like junctions so that distinct immunoreactive “hot spots” occur as separated bands at the corresponding levels. Close inspection of the syntaxin-4 staining, however, revealed that the two bands were not so clearly separated, as immunoreactivity could be found bridging the region between the two bands. Hirano and colleagues [57] showed in ultrastructural studies (their Fig. 6D) that syntaxin-4 was localized not only in the horizontal cell dendritic tips but also in the ascending dendrites. Here, the staining quality of parvalbumin at the ascending/invaginating horizontal cell dendrites was not sufficient to unambiguously confirm the notion, but we expect that the syntaxin-4 labeled “bridges” originate from these ascending horizontal cell dendrites also in primate.

### Syntaxin-4 is Enriched in HII Cells Beneath Primate S-cones

**Figure 2:**
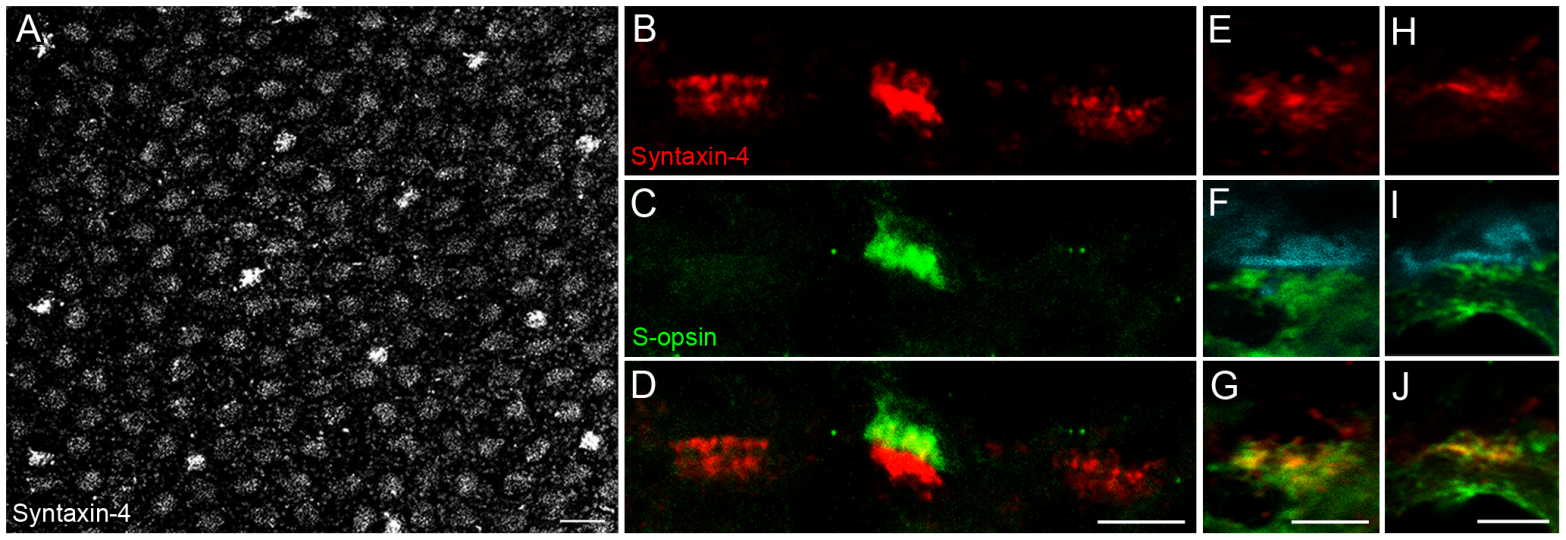
Syntaxin-4 is highly enriched beneath S-cone pedicles and colocalizes with HII horizontal cells. **A:** Labeling of syntaxin-4 in whole-mounted macaque retina at the level of the OPL. **B–D:** Staining of syntaxin-4 at three cone pedicles on a vertical cryostat section of macaque OPL. The section was double labeled with antibodies against S-opsin to identify S-cone pedicles. **E–J:** Single optical slices of vertical sections of macaque retina triple labeled against syntaxin-4 (red), S-opsin (cyan), and calbindin (CaBP, green), a marker for HII horizontal cells. Staining at two S-cone pedicles (E–G and H–J) is shown. Scale bars: 10 µm in A, 5 µm in D, G, J.

Our results substantiated findings from other species that all syntaxin-4 in the OPL is expressed by horizontal cells. Nevertheless, this conclusion could not explain the appearance of the large aggregates of the protein which also colocalized with parvalbumin. To further investigate these aggregates, whole-mounted macaque retina was labeled with an antibody against syntaxin-4 (Fig. 2A). The use of whole-mounted retina yielded an overview of a larger field of staining, where clusters of syntaxin-4 became apparent at all cone pedicles with intermingled puncta corresponding to staining at rod spherules. In addition, the dense aggregates occurred at multiple sites in a fairly regular mosaic, closely resembling the spatial distribution of S-cones in primate retina [79]. Indeed, double-labeling experiments revealed that syntaxin-4 was highly enriched at S-cone pedicles (Fig. 2B–D). The latter were identified by staining with antibodies against S-opsin, which label the complete cone photoreceptor cell including pedicle in primate retina [80]. The selective accumulation of syntaxin-4 at S-cones occurred primarily in the lower band. Taking the connectivity pattern of primate horizontal cells into account (see Introduction), our data strongly suggests that syntaxin-4 is enriched in HII horizontal cells at the level of their desmosome-like junctions beneath S-cones. To confirm this notion, vertical cryostat sections were triple immunolabeled with antibodies against syntaxin-4, S-opsin, and calbindin (CaBP) (Fig. 2E–J). In primate, CaBP labels HII horizontal cells in addition to cones and two types of diffuse bipolar cells [45]. At S-cones, the CaBP staining was concentrated beneath the pedicle base as a result of HII cell processes converging onto S-cone pedicles. There, syntaxin-4 and CaBP showed a complete overlap, indicating that the enhanced syntaxin-4 expression indeed originated from HII horizontal cells. This particular staining pattern was not observed at L/M-cone pedicles.

### Syntaxin-4 at S-cones of Mouse and Ground Squirrel

**Figure 3:**
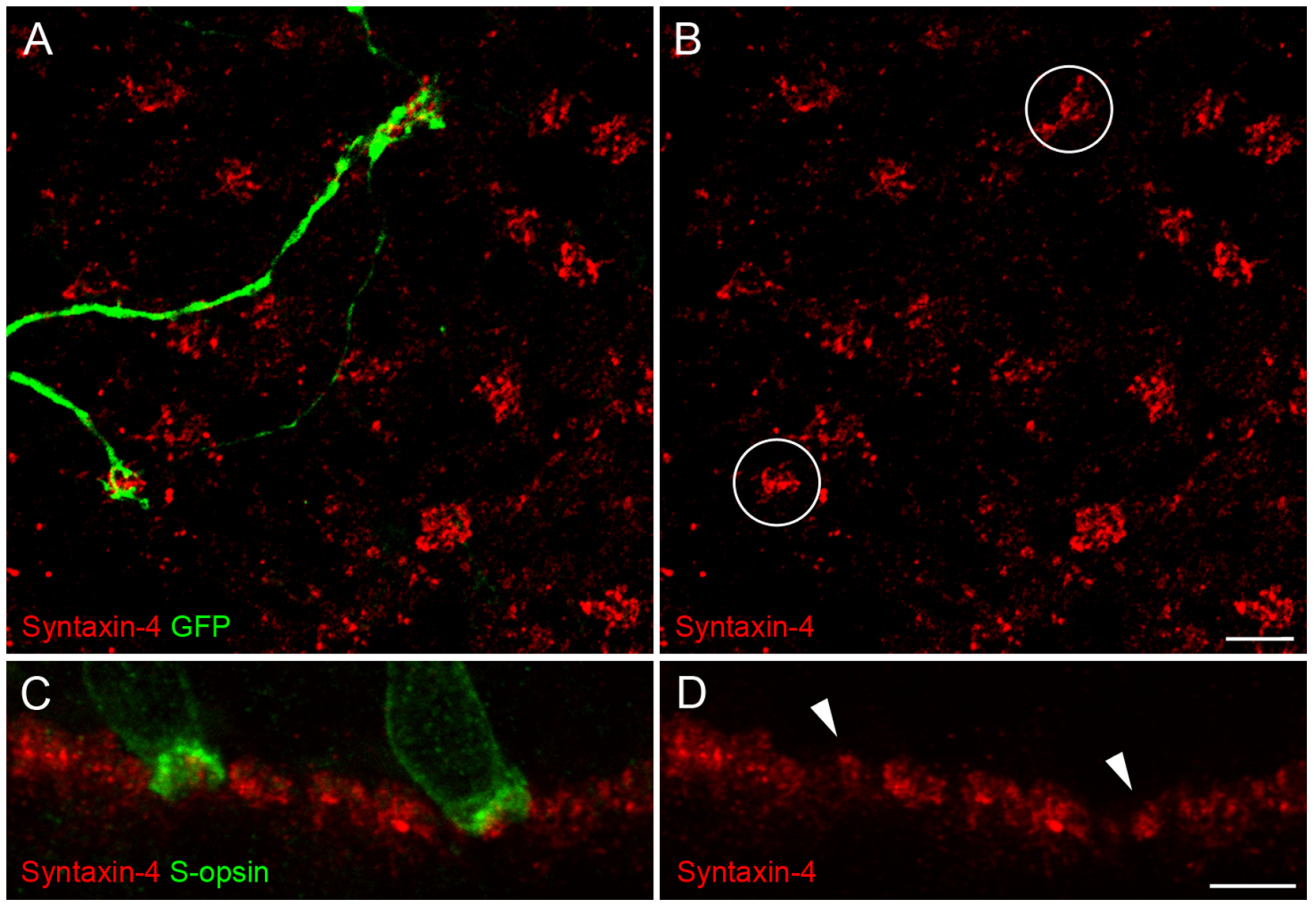
Syntaxin-4 is clustered but not enriched at S-cones of mouse and ground squirrel. **A, B:** Double labeling of syntaxin-4 and S-cone selective type-9 bipolar cells with antibodies against GFP on a horizontal section at the level of the OPL of Clm1 mouse retinas. **C, D:** Double labeling of syntaxin-4 and S-opsin on a vertical section of ground squirrel OPL. Circles in B and arrowheads in D indicate the position of S-cone pedicles. Scale bars: 5 µm.

The question arising from our findings was then concerned with the trigger of syntaxin-4 enrichment. Is the enhanced expression level of syntaxin-4 a common feature of the mammalian S-cone postsynapse, specific to S-cone preferring horizontal cells, or a unique property of primate HII horizontal cells? Mouse and ground squirrel retinas were used as models for different mammalian horizontal cell systems. Similar to primate and other mammals, mice possess “true blue” cones, which express S-opsin only [61]. However, they only have B-type horizontal cells, which most likely form non-selective contacts with all cones types [44], [81]. Figure 3A,B shows that syntaxin-4 was clustered at S-cones of mouse retina but not enriched in comparison to the other cone types. The location of S-cone pedicles was identified by dendritic endings of S-cone selective type 9 bipolar cells in the Clm1 mouse retina [61]. In contrast to mouse, ground squirrel horizontal cells comprise two types, with H2 (or A-type) horizontal cells being S-cone selective [54], [55]. Here, S-cones of ground squirrel retina could again be labeled with antibodies against S-opsin [82]. A counterstain with syntaxin-4 revealed that the latter was expressed beneath S-cones, mostly at lower levels when compared to the remaining cones (Fig. 3C,D). Taken together, syntaxin-4 expression levels at S-cones did not exceed the protein density at other cone types in both mouse and ground squirrel retinas. Therefore, the enrichment of syntaxin-4 beneath primate S-cones is likely a unique feature of primate HII horizontal cells.

### Spatial Distribution of Syntaxin-4 and GABA-R in Primate OPL

**Figure 4:**
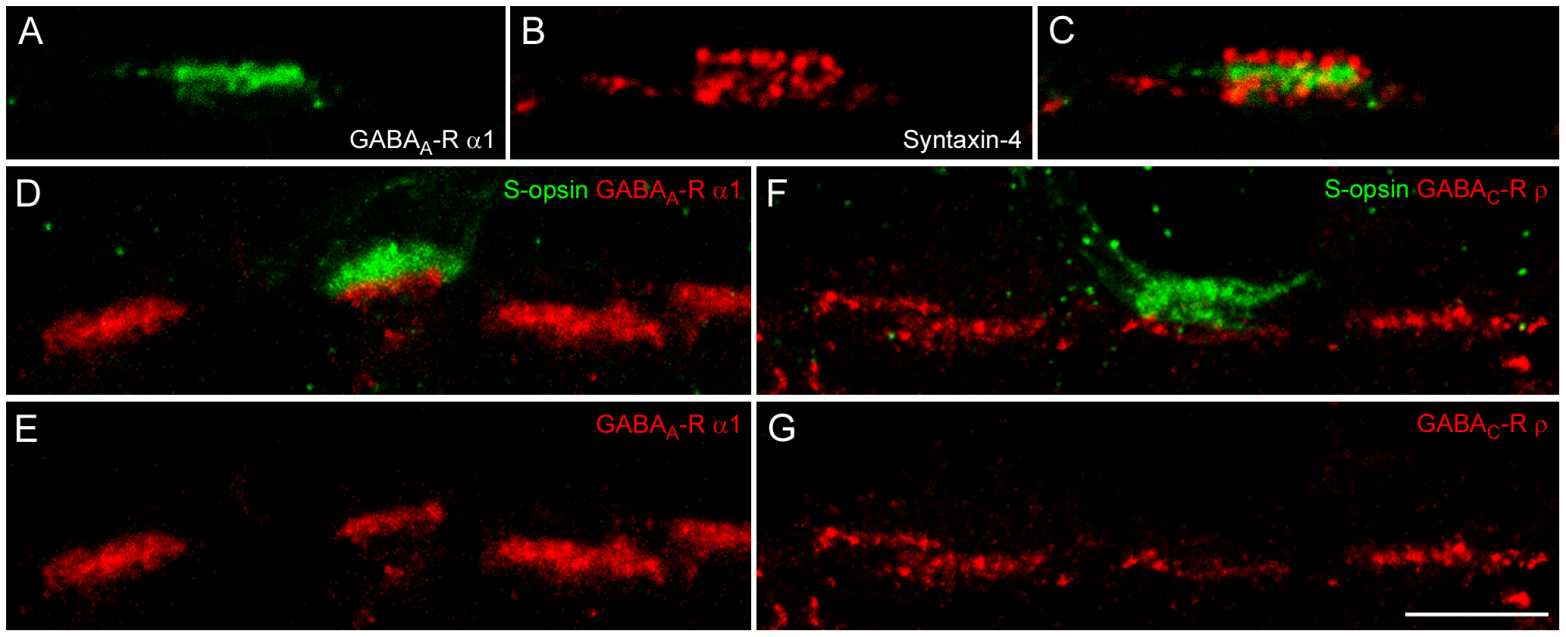
GABA receptors and syntaxin-4 in primate OPL. **A–C:** Double labeling of syntaxin-4 and GABA_A_ receptor subunits α1 on a vertical cryostat section of macaque OPL. **D–G:** Double labeling of S-cone pedicles with S-opsin and either GABA_A_-R α1 (D, E) or GABA_C_-R ρ subunits (F, G). Scale bar: 5 µm, applies to all images.

As mentioned earlier, syntaxin-4 may indicate GABA release from horizontal cells. In line with this idea, further essential elements are required on the postsynaptic side to have GABA release elicit its effects, such as GABA receptors. The GABA receptors in the OPL of macaque retina were tightly sandwiched between the two syntaxin-4 bands (Fig. 4A–C), a spatial organization which would allow GABA to take immediate effect. The finding of enhanced expression levels of syntaxin-4 at primate S-cones points to interesting functional consequences for the S-cone pathway as discussed in the next section. However, the expression pattern of GABA receptors with regard to the S-cone pathway has not been analyzed, yet. Thus, double labeling experiments of S-cones and the two of the major GABA receptor subunits expressed in primate OPL [20] were performed. Both GABA_A_-R α1 and GABA_C_-R ρ subunits were found beneath S-cone pedicles (Fig. 4D–G).

### Chloride Transporters Beneath Primate S-cones

**Figure 5:**
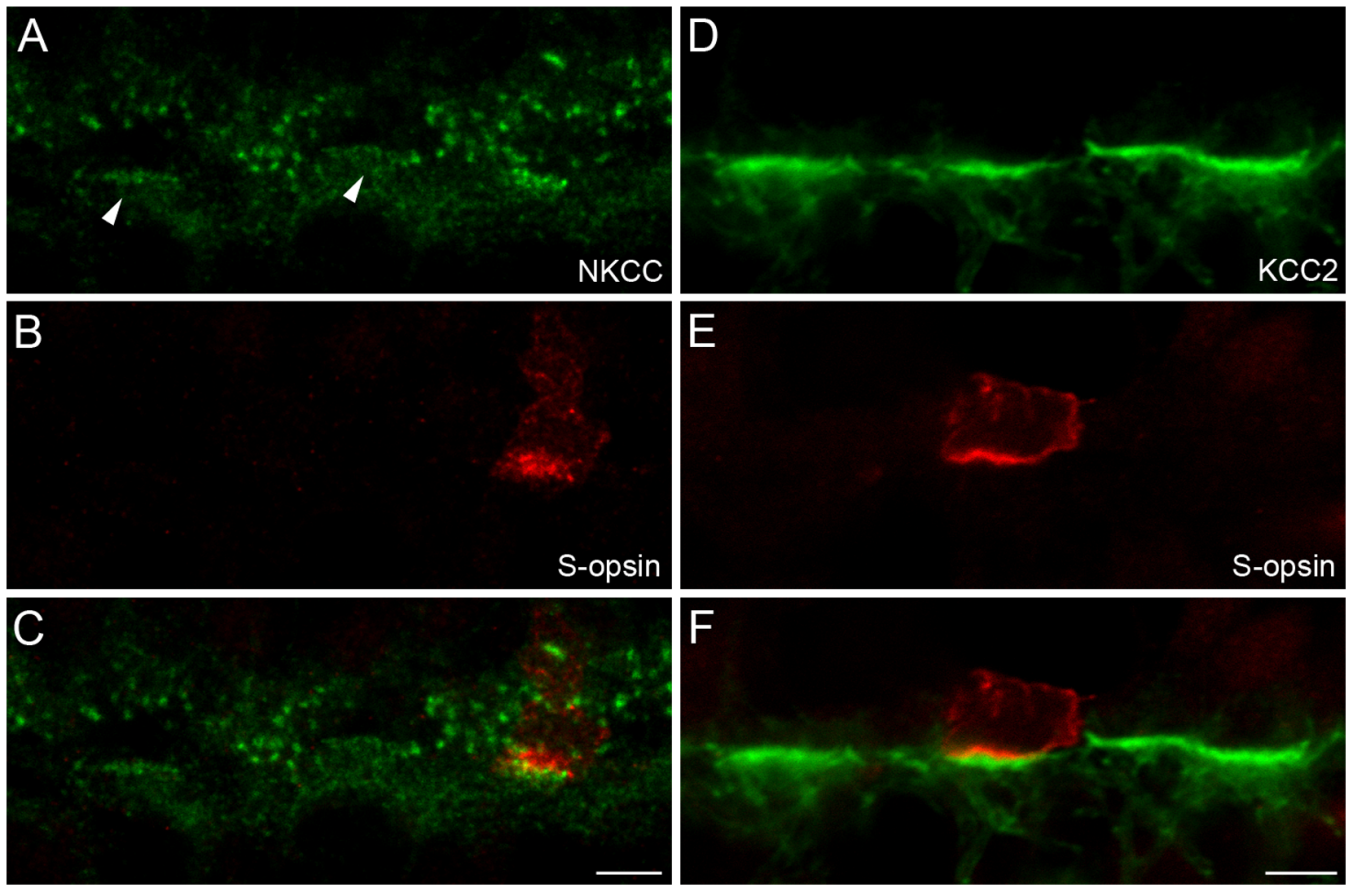
Staining of NKCC and KCC2 at S-cone pedicles in comparison to L/M-cones. Labeling of NKCC (**A–C**) and KCC2 (**D–F**)**,** each counterstained with antibodies against S-opsin, on vertical cryosections of macaque OPL. Arrowheads point to NKCC and KCC2 expression beneath L/M-cones. Scale bar: 5 µm.

In addition, macaque retina was labeled against S-opsin and the chloride transporters NKCC and KCC2 to investigate the expression pattern of another crucial component in a GABAergic signaling pathway at this site. The overall staining patterns resembled the data published by Vardi and colleagues [38]. There, a rather diffuse staining of NKCC at cone pedicles and a brighter, more punctate appearance postsynaptic to rods was shown. Here, however, a fundamental difference of NKCC expression at L/M- versus S-cone pedicles became obvious Fig. 5A–C). In contrast to the diffusely labeled L/M-cones (indicated by arrowheads), clusters of discrete NKCC puncta were observed at S-cones, similar to the distinct aggregates at rods.

Staining of KCC2 revealed no obvious differences in the expression pattern of this chloride transporter subunit when L/M- and S-cones were compared (Fig. 5D–F). Dense labeling of KCC2 in putative OFF bipolar cell dendrites occurred beneath all cone pedicles.

## Discussion

In this study, we investigated the expression patterns of different proteins in the outer mammalian retina, which may be involved in a GABAergic signaling cascade between horizontal cells and bipolar cells. We showed that the overall expression pattern of syntaxin-4 in macaque retina closely resembles findings from other species [15], [57], [59] indicating a conserved role of this protein in mammalian retinas. Syntaxin-4 was enhanced in HII cells and therefore highly enriched beneath S-cones, a phenomenon which was not seen in mouse or ground squirrel retinas. Furthermore, we provided evidence that other key elements for GABAergic feed-forward signal transmission are expressed beneath primate S-cones, such as GABA receptors and high amounts of the chloride transporters NKCC and KCC2.

### Syntaxin-4 and Chloride Transporters Beneath S-cones

The results presented here, combined with the known photoreceptor-connectivity pattern of primate horizontal cells, allow drawing definite conclusions about syntaxin-4 expression in the outer retina. Syntaxin-4 was colocalized with horizontal cell processes postsynaptic to rods and to all cone types, while it was highly enriched in HII horizontal cells beneath S-cones. HI horizontal cells contact rods as well as L/M-cones but avoid contacts with S-cones. In contrast, HII horizontal cells are engaged with multiple S-cones and make sparser connections with L/M-cone pedicles [49], [50]. Therefore, syntaxin-4 appears to be expressed by both horizontal cell types, but its expression is enhanced in HII horizontal cells.

Functional data describing the role of syntaxin-4 in mammalian horizontal cells is still missing and very little is known about this particular syntaxin isoform in neuronal cells altogether. Evidence exists that syntaxin-4 is involved in exocytosis of AMPA-type glutamate receptor containing vesicles in hippocampal neurons [83]. This particular role is a possibility in retinal neurons but it seems unlikely, especially with regard to horizontal cells. In contrast to what we have shown here for syntaxin-4, an enhanced expression of horizontal cell glutamate receptors at primate S-cone pedicles in comparison with L/M-cones does not occur [69], [84]–[86]. Recently, it has been hypothesized that synytaxin-4 indicates GABA release from mammalian horizontal cells, based on the facts that horizontal cells contain GABA and express the required protein machinery for exocytotic activity, including syntaxin-4, SNAP-25, and synaptotagmin-2 [15], [57]. Assuming that syntaxin-4 is involved in transmitter release from these cells, the elevated staining levels of syntaxin-4 at S-cones presented herein would be consistent with a proposal of GABA release via HII horizontal cells at primate S-cones. The denser expression pattern of the chloride transporter NKCC at S-cones in comparison to L/M-cones lines up perfectly with this idea, as this seems to be a postsynaptic specification in response to the transmitter release. It was shown that NKCC is expressed in dendritic tips of ON bipolar cells, where it elicits an excitatory effect of GABA due to chloride accumulation [38], [40]–[42]. Furthermore, it is well-known that the blue cone bipolar cell is the major postsynaptic ON bipolar cell type of S-cones in various species including primate [54], [76], [87]–[89]. In summary, our findings suggest that there is a possibility of GABA release via HII horizontal cells beneath S-cones. At all cone types, GABA would elicit the expected inhibitory effect on OFF bipolar cells based on the dense expression of KCC2. However, the same neurotransmitter would cause an excitatory effect in ON bipolar cells. This GABA-mediated excitation is particularly strong in blue cone bipolar cells of mouse retina [40] and may also exist in primate blue cone bipolar cells as suggested by the expression of NKCC beneath primate S-cones (shown herein). This kind of signal transmission makes intriguing predictions about its functional role in the S-cone pathway of primate retina – as it may largely contribute to the transmission of yellow-OFF signals via blue cone bipolar cells.

### Pathways for Yellow-OFF Signals

**Figure 6:**
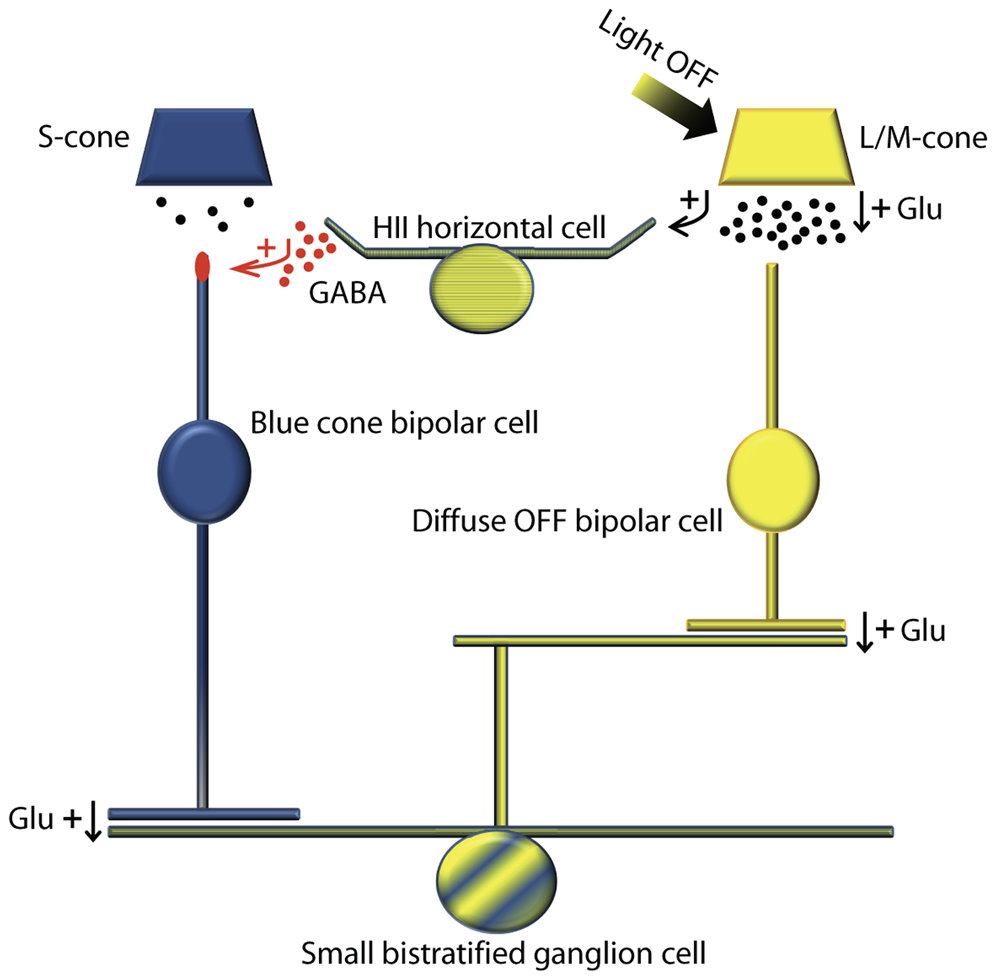
Pathways of synaptic transmission causing blue-ON vs. yellow-OFF light responses in small bistratified ganglion cells. Red labels indicate a pathway where GABA is thought to elicit a strong depolarization in blue cone bipolar cells according to high expression levels of NKCC on their dendritic tips. Arrows indicate excitatory (+) synaptic transmission via GABA or glutamate (Glu). See Discussion for a detailed description.

Yellow-OFF signals are known to be directly conveyed to the IPL via the canonical glutamatergic pathway of diffuse OFF bipolar cells contacting L/M-cones. Here, based on our anatomical data, we propose an additional synaptic pathway which may function to transmit L/M-OFF signals to the inner retina (Fig. 6). At the offset of yellow light, L- and M-cones depolarize and release increased amounts of glutamate, which in turn leads to the depolarization of HII horizontal cells. As suggested by syntaxin-4 accumulation, HII cells would then release large quantities of GABA beneath S-cones onto blue cone bipolar cells. This would elicit a chloride efflux from dendrites of blue cone bipolar cells because of high levels of NKCC, causing a strong depolarization in this cell type and, in turn, contribute to the yellow-OFF response in the small bistratified ganglion cell [90].

The small bistratified ganglion cell receives S-cone input via blue cone (ON) bipolar cell synapses onto its inner dendritic tier. Additionally, diffuse OFF bipolar cells transmit L/M-cone signals and synapse onto the outer tier [89], [91], [92]. Thereby, the small bistratified ganglion cell carries color-opponent blue-ON/yellow-OFF excitatory signals [90]. Crook and colleagues [93] provided functional evidence for direct excitatory OFF bipolar cell input to the small bistratified ganglion cell. However, data regarding the spatial structure and synaptic origins of this ganglion cell’s receptive field remain controversial [94], [95]. Interestingly, the yellow-OFF response of small bistratified ganglion cells was delayed when compared to the blue-ON response, and largely abolished by a pharmacological block of all ON bipolar cells [94]. These findings lead the authors to speculate that the yellow-OFF response is transmitted in large part via blue cone (ON) bipolar cells and originates from horizontal cells feeding L/M-cone signals back to the S-cones.

The hypothesized feed-forward pathway also makes sense from an anatomical point of view. Although the aforementioned evidence for a canonical yellow-OFF pathway via diffuse OFF bipolar cells appears solid, morphological details of small bistratified ganglion cells seem to require additional yellow-OFF routes. Yellow-OFF signals are robust and usually balanced in strength when compared with blue-ON responses across the full range of retinal eccentricity. However, the outer dendritic tier is often very sparse in comparison to the inner at more peripheral eccentricities, and relatively few OFF bipolar cells synapses were observed on these cells [91], [96]. A yellow-OFF pathway via HII and blue cone bipolar cells could compensate for the reduction of the canonical OFF bipolar cell input to the small bistratified cell in more peripheral regions of the retina. Therefore, it may help to explain constancy of the response pattern despite morphological changes of the outer dendrites across the retina.Pathways for spectrally opponent responses also exist in non-primate mammals (reviewed in [54]. In contrast to primate, we did not find an enriched expression of syntaxin-4 at S-cones in these species. Nevertheless, the effect of chloride accumulation in dendrites of ON bipolar cells and the resulting depolarizing responses to GABA were strongest in type 9 bipolar cells of the mouse retina [40], which has been shown to be the S-cone selective (blue) cone bipolar cell [61]. Therefore, the kind of GABAergic feed-forward excitation within the S-cone opponent circuitry we propose here may very likely exist also in other species, such as mice, but the enhancement of syntaxin-4 expression at S-cones relative to other cone types appears to be a primate-specific phenomenon.

### GABA Release from Horizontal Cells

The fact that the pathway described herein is partially based on GABA may help explain some of the controversial results regarding the receptive field structure of small bistratified ganglion cells. As mentioned above, horizontal cells have been found to be GABA-positive in many species. However, immunostainings are often erratic or even negative. In line with these anatomical observations, functional analyses of retinal in vitro preparations indicated that GABA had no major effect on the receptive field structure and the surround of primate ganglion cells [93], [97]. GABA release from horizontal cells upon depolarization has been shown in fish [98], and the existence of GABA as well as the required release machinery in mammalian horizontal cells has been demonstrated before (see Introduction). But why does GABAergic signaling in the outer mammalian retina often remain elusive and functional evidence even absent? A first hint to solve this mystery has already been provided almost two decades ago. Pow and colleagues [99] investigated the uptake of GABA analogues into retinal neurons of various mammalian and non-mammalian species. They found that GABA uptake by horizontal cells strongly differs between species and experimental conditions, e.g. the adaptational state of the retina, while cat and monkey horizontal cells appeared to lack any uptake mechanism altogether. The fact that cat and monkey horizontal cells have been found GABA-immunoreactive [18], [19], [25]–[27] indicates that these cells do contain GABA originally but “lose” their neurotransmitter depending on experimental conditions in the respective studies. In line with this notion, it has been hypothesized that GABA in primate horizontal cells may be extremely volatile [19] and may undergo fast post-mortem degradation [17]. Recently, this issue was directly addressed in mouse retina [23]. There, GABA-immunoreactivity of horizontal cells was restored in vitro by supplementing common incubation media with the GAD cofactor pyridoxal 5′-phosphate, and GABA restoration was further enhanced by increased levels of the GAD substrate glutamate (or glutamine).

Taken together, horizontal cells apparently lack a sufficient re-uptake mechanism for GABA in many if not all mammalian species and continuously synthesize this transmitter in vivo at presumably high rates. GABA synthesis then gets disturbed or completely stalled after the tissue is harvested from the animal. These features contrast those of most other neurons and may explain the erratic and often controversial anatomical as well as functional results.

## Supporting Information

Checklist S1 ARRIVE Guidelines Checklist.
(DOC)

## References

[1] Baylor DA, Fuortes MG, O’Bryan PM (1971) Receptive fields of cones in the retina of the turtle. J Physiol 214: 265–294. 5579638 10.1113/jphysiol.1971.sp009432 PMC1331836

[2] Verweij J, Hornstein EP, Schnapf JL (2003) Surround antagonism in macaque cone photoreceptors. J Neurosci 23: 10249–10257. 14614083 10.1523/JNEUROSCI.23-32-10249.2003 PMC6741006

[3] Verweij J, Kamermans M, Spekreijse H (1996) Horizontal cells feed back to cones by shifting the cone calcium-current activation range. Vision Res 36: 3943–3953. 9068848 10.1016/s0042-6989(96)00142-3

[4] Packer OS, Verweij J, Li PH, Schnapf JL, Dacey DM (2010) Blue-yellow opponency in primate S cone photoreceptors. J Neurosci 30: 568–572. 20071519 10.1523/JNEUROSCI.4738-09.2010 PMC2826135

[5] Dacey DM, Packer OS, Diller LC, Brainard DH, Peterson BB, et al (2000) Center surround receptive field structure of cone bipolar cells in primate retina. Vision Res 40: 1801–1811. 10837827 10.1016/s0042-6989(00)00039-0

[6] Kaneko A (1973) Receptive field organization of bipolar and amacrine cells in the goldfish retina. JPhysiol (Lond) 235: 133–153. 4778132 10.1113/jphysiol.1973.sp010381 PMC1350736

[7] Dacheux RF, Miller RF (1981) An intracellular electrophysiological study of the ontogeny of functional synapses in the rabbit retina. I. Receptors, horizontal, and bipolar cells. J Comp Neurol 198: 307–326. 7240448 10.1002/cne.901980209

[8] Fahey PK, Burkhardt DA (2003) Center-surround organization in bipolar cells: symmetry for opposing contrasts. Vis Neurosci 20: 1–10. 12699078 10.1017/s0952523803201012

[9] Kuffler SW (1953) Discharge patterns and functional organization of mammalian retina. J Neurophysiol 16: 37–68. 13035466 10.1152/jn.1953.16.1.37

[10] Barlow HB, Fitzhugh R, Kuffler SW (1957) Change of organization in the receptive fields of the cat’s retina during dark adaptation. J Physiol 137: 338–354. 13463771 10.1113/jphysiol.1957.sp005817 PMC1363009

[11] Enroth-Cugell C, Lennie P (1975) The control of retinal ganglion cell discharge by receptive field surrounds. JPhysiol (Lond) 247: 551–578. 1142301 10.1113/jphysiol.1975.sp010947 PMC1309488

[12] Davenport CM, Detwiler PB, Dacey DM (2008) Effects of pH buffering on horizontal and ganglion cell light responses in primate retina: evidence for the proton hypothesis of surround formation. J Neurosci 28: 456–464. 18184788 10.1523/JNEUROSCI.2735-07.2008 PMC3057190

[13] Picaud S, Pattnaik B, Hicks D, Forster V, Fontaine V, et al (1998) GABA_A_ and GABAC receptors in adult porcine cones: evidence from a photoreceptor-glia co-culture model. JPhysiol (Lond) 513: 33–42. 9782157 10.1111/j.1469-7793.1998.033by.x PMC2231280

[14] Kaneko A, Tachibana M (1986) Effects of gamma-aminobutyric acid on isolated cone photoreceptors of the turtle retina. J Physiol 373: 443–461. 3746679 10.1113/jphysiol.1986.sp016057 PMC1182547

[15] Lee H, Brecha NC (2010) Immunocytochemical evidence for SNARE protein-dependent transmitter release from guinea pig horizontal cells. Eur J Neurosci 31: 1388–1401. 20384779 10.1111/j.1460-9568.2010.07181.x PMC3743676

[16] Guo C, Hirano AA, Stella SL Jr, Bitzer M, Brecha NC (2010) Guinea pig horizontal cells express GABA, the GABA-synthesizing enzyme GAD 65, and the GABA vesicular transporter. J Comp Neurol 518: 1647–1669. 20235161 10.1002/cne.22294 PMC3736838

[17] Jellali A, Stussi-Garaud C, Gasnier B, Rendon A, Sahel JA, et al (2002) Cellular localization of the vesicular inhibitory amino acid transporter in the mouse and human retina. J Comp Neurol 449: 76–87. 12115694 10.1002/cne.10272

[18] Kao Y, Lassova L, Bar-Yehuda T, Edwards R, Sterling P, et al (2004) Evidence that certain retinal bipolar cells use both glutamate and GABA. J Comp Neurol 478: 207–218. 15368537 10.1002/cne.20221

[19] Kalloniatis M, Marc RE, Murry RF (1996) Amino acid signatures in the primate retina. JNeurosci 16: 6807–6829. 8824321 10.1523/JNEUROSCI.16-21-06807.1996 PMC6579280

[20] Haverkamp S, Grünert U, Wässle H (2000) The cone pedicle, a complex synapse in the retina. Neuron 27: 85–95. 10939333 10.1016/s0896-6273(00)00011-8

[21] Cueva JG, Haverkamp S, Reimer RJ, Edwards R, Wässle H, et al (2002) Vesicular gamma-aminobutyric acid transporter expression in amacrine and horizontal cells. J Comp Neurol 445: 227–237. 11920703 10.1002/cne.10166 PMC3696019

[22] Hirano AA, Brandstatter JH, Morgans CW, Brecha NC (2011) SNAP25 expression in mammalian retinal horizontal cells. J Comp Neurol 519: 972–988. 21280047 10.1002/cne.22562 PMC4125362

[23] Deniz S, Wersinger E, Schwab Y, Mura C, Erdelyi F, et al (2011) Mammalian retinal horizontal cells are unconventional GABAergic neurons. J Neurochem 116: 350–362. 21091475 10.1111/j.1471-4159.2010.07114.x

[24] Herrmann R, Heflin SJ, Hammond T, Lee B, Wang J, et al (2011) Rod vision is controlled by dopamine-dependent sensitization of rod bipolar cells by GABA. Neuron 72: 101–110. 21982372 10.1016/j.neuron.2011.07.030 PMC3197016

[25] Wässle H, Chun MH (1989) GABA-like immunoreactivity in the cat retina: Light microscopy. JCompNeurol 279: 43–54. 10.1002/cne.902790105 2913060

[26] Agardh E, Ehinger B, Wu J-Y (1987) GABA and GAD-like immunoreactivity in the primate retina. Histochemistry 86: 485–490. 3294761 10.1007/BF00500621

[27] Grünert U, Wässle H (1990) GABA-like immunoreactivity in the macaque monkey retina: A light and electron microscopic study. JCompNeurol 297: 509–524. 10.1002/cne.902970405 2384611

[28] Thoreson WB, Burkhardt DA (1990) Effects of synaptic blocking agents on the depolarizing responses of turtle cones evoked by surround illumination. VisNeurosci 5: 571–583. 10.1017/s0952523800000730 2085473

[29] Klaassen LJ, Fahrenfort I, Kamermans M (2012) Connexin hemichannel mediated ephaptic inhibition in the retina. Brain Res 1487: 25–38. 22796289 10.1016/j.brainres.2012.04.059

[30] Hirasawa H, Yamada M, Kaneko A (2012) Acidification of the synaptic cleft of cone photoreceptor terminal controls the amount of transmitter release, thereby forming the receptive field surround in the vertebrate retina. J Physiol Sci 62: 359–375. 22773408 10.1007/s12576-012-0220-0 PMC10717482

[31] Dowling JE, Brown JE, Major D (1966) Synapses of horizontal cells in rabbit and cat retinas. Science 153: 1639–1641. 5917075 10.1126/science.153.3744.1639

[32] Kolb H (1977) The organization of the outer plexiform layer in the retina of the cat: electron microscopic observations. J Neurocytol 6: 131–153. 856949 10.1007/BF01261502

[33] Fisher SK, Boycott BB (1974) Synaptic connections made by horizontal cells within the outer plexiform layer of the retina of the cat and the rabbit. Proc R Soc Lond B Biol Sci 186: 317–331. 4154444 10.1098/rspb.1974.0052

[34] Missotten L (1965) The Ultrastructure of the Human Retina. Brussels: Editions Arscia SA.

[35] Dowling JE, Boycott BB (1966) Organization of the primate retina: electron microscopy. Proc R Soc Lond B Biol Sci 166: 80–111. 4382694 10.1098/rspb.1966.0086

[36] Puller C, de Sevilla Müller LP, Janssen-Bienhold U, Haverkamp S (2009) ZO-1 and the spatial organization of gap junctions and glutamate receptors in the outer plexiform layer of the mammalian retina. J Neurosci 29: 6266–6275. 19439604 10.1523/JNEUROSCI.5867-08.2009 PMC6665500

[37] Vardi N, Sterling P (1994) Subcellular localization of GABA_A_ receptor on bipolar cells in macaque and human retina. Vision Res 34: 1235–1246. 8023433 10.1016/0042-6989(94)90198-8

[38] Vardi N, Zhang LL, Payne JA, Sterling P (2000) Evidence that different cation chloride cotransporters in retinal neurons allow opposite responses to GABA. J Neurosci 20: 7657–7663. 11027226 10.1523/JNEUROSCI.20-20-07657.2000 PMC6772883

[39] Miller RF, Dacheux RF (1983) Intracellular chloride in retinal neurons: measurement and meaning. Vision Res 23: 399–411. 6880038 10.1016/0042-6989(83)90087-1

[40] Duebel J, Haverkamp S, Schleich W, Feng G, Augustine GJ, et al (2006) Two-photon imaging reveals somatodendritic chloride gradient in retinal ON-type bipolar cells expressing the biosensor Clomeleon. Neuron 49: 81–94. 16387641 10.1016/j.neuron.2005.10.035

[41] Varela C, Blanco R, De la Villa P (2005) Depolarizing effect of GABA in rod bipolar cells of the mouse retina. Vision Res 45: 2659–2667. 15923018 10.1016/j.visres.2005.03.020

[42] Chaffiol AJ, Cao Y, Ishii M, Ribelayga C, Mangel SC (2012) Light/Dark Adaptive Regulation of GABAA Receptor and NKCC Expression and Activity Modulates Direct, GABA-mediated Horizontal Cell Signaling to ON-Cone Bipolar Cells. Invest Ophthalmol Vis Sci 53: 4306.22661482

[43] Chiang PH, Wu PY, Kuo TW, Liu YC, Chan CF, et al (2012) GABA is depolarizing in hippocampal dentate granule cells of the adolescent and adult rats. J Neurosci 32: 62–67. 22219270 10.1523/JNEUROSCI.3393-11.2012 PMC6621339

[44] Peichl L (2010) Morphology of Interneurons: Horizontal Cells. DA Dartt, editor Encyclopedia of the Eye, Vol 3 Oxford: Academic Press.

[45] Wässle H, Dacey DM, Haun T, Haverkamp S, Grünert U, et al (2000) The mosaic of horizontal cells in the macaque monkey retina: with a comment on biplexiform ganglion cells. Vis Neurosci 17: 591–608. 11016578 10.1017/s0952523800174097

[46] Wässle H, Boycott BB, Röhrenbeck J (1989) Horizontal Cells in the Monkey Retina: Cone connections and dendritic network. Eur J Neurosci 1: 421–435. 12106129 10.1111/j.1460-9568.1989.tb00350.x

[47] Ahnelt P, Kolb H (1994) Horizontal cells and cone photoreceptors in primate retina: A golgi-light microscopic study of spectral connectivity. JCompNeurol 343: 387–405. 10.1002/cne.903430305 8027449

[48] Ahnelt P, Kolb H (1994) Horizontal cells and cone photoreceptors in human retina: A golgi-electron microscopic study of spectral connectivity. JCompNeurol 343: 406–427. 10.1002/cne.903430306 8027450

[49] Dacey DM, Lee BB, Stafford DK, Pokorny J, Smith VC (1996) Horizontal cells of the primate retina: cone specificity without spectral opponency. Science 271: 656–659. 8571130 10.1126/science.271.5249.656

[50] Goodchild AK, Chan TL, Grünert U (1996) Horizontal cell connections with short-wavelength-sensitive cones in macaque monkey retina. Vis Neurosci 13: 833–845. 8903027 10.1017/s0952523800009093

[51] Chan TL, Grünert U (1998) Horizontal cell connections with short wavelength-sensitive cones in the retina: A comparison between New World and Old World primates. JCompNeurol 393: 196–209. 9548697

[52] Kolb H, Mariani A, Gallego A (1980) A second type of horizontal cell in the monkey retina. JCompNeurol 189: 31–44. 10.1002/cne.901890103 6766145

[53] Sandmann D, Boycott BB, Peichl L (1996) Blue cone horizontal cells in the retinae of horses and other *equidae.* . JNeurosci 16: 3381–3396. 8627374 10.1523/JNEUROSCI.16-10-03381.1996 PMC6579128

[54] Puller C, Haverkamp S (2011) Bipolar cell pathways for color vision in non-primate dichromats. Vis Neurosci 28: 51–60. 21070688 10.1017/S0952523810000271

[55] Linberg KA, Suemune S, Fisher SK (1996) Retinal neurons of the California ground squirrel, *Spermophilus beecheyi*: A golgi study. JCompNeurol 365: 173–216. 10.1002/(SICI)1096-9861(19960205)365:2<173::AID-CNE1>3.0.CO;2-2 8822165

[56] Crook JD, Manookin MB, Packer OS, Dacey DM (2011) Horizontal cell feedback without cone type-selective inhibition mediates “red-green” color opponency in midget ganglion cells of the primate retina. J Neurosci 31: 1762–1772. 21289186 10.1523/JNEUROSCI.4385-10.2011 PMC3074339

[57] Hirano AA, Brandstatter JH, Vila A, Brecha NC (2007) Robust syntaxin-4 immunoreactivity in mammalian horizontal cell processes. Vis Neurosci 24: 489–502. 17640443 10.1017/S0952523807070198 PMC2744743

[58] Südhof TC, Rizo J (2011) Synaptic vesicle exocytosis. Cold Spring Harb Perspect Biol 3. 10.1101/cshperspect.a005637 PMC3225952 22026965

[59] Sherry DM, Mitchell R, Standifer KM, du Plessis B (2006) Distribution of plasma membrane-associated syntaxins 1 through 4 indicates distinct trafficking functions in the synaptic layers of the mouse retina. BMC Neurosci 7: 54. 16839421 10.1186/1471-2202-7-54 PMC1555595

[60] Berglund K, Schleich W, Krieger P, Loo LS, Wang D, et al (2006) Imaging synaptic inhibition in transgenic mice expressing the chloride indicator, Clomeleon. Brain Cell Biol 35: 207–228. 18398684 10.1007/s11068-008-9019-6 PMC2673725

[61] Haverkamp S, Wässle H, Duebel J, Kuner T, Augustine GJ, et al (2005) The primordial, blue-cone color system of the mouse retina. J Neurosci 25: 5438–5445. 15930394 10.1523/JNEUROSCI.1117-05.2005 PMC6725002

[62] Benke D, Mertens S, Trzeciak A, Gillessen D, Mohler H (1991) GABAA receptors display association of gamma 2-subunit with alpha 1- and beta 2/3-subunits. J Biol Chem 266: 4478–4483. 1847922

[63] Schoch P, Richards JG, Haring P, Takacs B, Stahli C, et al (1985) Co-localization of GABA receptors and benzodiazepine receptors in the brain shown by monoclonal antibodies. Nature 314: 168–171. 2983231 10.1038/314168a0

[64] Enz R, Brandstätter JH, Wässle H, Bormann J (1996) Immunocytochemical localization of the GABA_c_ receptor rho subunits in the mammalian retina. JNeurosci 16: 4479–4490. 8699258 10.1523/JNEUROSCI.16-14-04479.1996 PMC6578859

[65] Delgado LM, Vielma AH, Kahne T, Palacios AG, Schmachtenberg O (2009) The GABAergic system in the retina of neonate and adult Octodon degus, studied by immunohistochemistry and electroretinography. J Comp Neurol 514: 459–472. 19350652 10.1002/cne.22023

[66] Wässle H, Puller C, Müller F, Haverkamp S (2009) Cone contacts, mosaics, and territories of bipolar cells in the mouse retina. J Neurosci 29: 106–117. 19129389 10.1523/JNEUROSCI.4442-08.2009 PMC6664901

[67] Breuninger T, Puller C, Haverkamp S, Euler T (2011) Chromatic bipolar cell pathways in the mouse retina. J Neurosci 31: 6504–6517. 21525291 10.1523/JNEUROSCI.0616-11.2011 PMC6622657

[68] Dumitrescu ON, Pucci FG, Wong KY, Berson DM (2009) Ectopic retinal ON bipolar cell synapses in the OFF inner plexiform layer: contacts with dopaminergic amacrine cells and melanopsin ganglion cells. J Comp Neurol 517: 226–244. 19731338 10.1002/cne.22158 PMC3296562

[69] Puller C, Haverkamp S (2011) Cell-type-specific localization of protocadherin beta16 at AMPA and AMPA/Kainate receptor-containing synapses in the primate retina. J Comp Neurol 519: 467–479. 21192079 10.1002/cne.22528

[70] Lytle C, Xu JC, Biemesderfer D, Forbush B 3rd (1995) Distribution and diversity of Na-K-Cl cotransport proteins: a study with monoclonal antibodies. Am J Physiol 269: C1496–1505. 8572179 10.1152/ajpcell.1995.269.6.C1496

[71] Zhang LL, Fina ME, Vardi N (2006) Regulation of KCC2 and NKCC during development: membrane insertion and differences between cell types. J Comp Neurol 499: 132–143. 16958091 10.1002/cne.21100

[72] Li B, McKernan K, Shen W (2008) Spatial and temporal distribution patterns of Na-K-2Cl cotransporter in adult and developing mouse retinas. Vis Neurosci 25: 109–123. 18442435 10.1017/S0952523808080164 PMC5531596

[73] Zhang LL, Delpire E, Vardi N (2007) NKCC1 does not accumulate chloride in developing retinal neurons. J Neurophysiol 98: 266–277. 17493914 10.1152/jn.00288.2007

[74] Williams JR, Sharp JW, Kumari VG, Wilson M, Payne JA (1999) The neuron-specific K-Cl cotransporter, KCC2. Antibody development and initial characterization of the protein. J Biol Chem 274: 12656–12664. 10212246 10.1074/jbc.274.18.12656

[75] MacNeil MA, Gaul PA (2008) Biocytin wide-field bipolar cells in rabbit retina selectively contact blue cones. J Comp Neurol 506: 6–15. 17990268 10.1002/cne.21491 PMC2838454

[76] Puller C, Ondreka K, Haverkamp S (2011) Bipolar cells of the ground squirrel retina. J Comp Neurol 519: 759–774. 21246553 10.1002/cne.22546

[77] Röhrenbeck J, Wässle H, Boycott BB (1989) Horizontal cells in the monkey retina: immunocytochemical staining with antibodies against calcium binding proteins. EurJNeurosci 1: 407–420. 10.1111/j.1460-9568.1989.tb00349.x 12106128

[78] Chiquet C, Dkhissi-Benyahya O, Cooper H (2005) Calcium-binding protein distribution in the retina of strepsirhine and haplorhine primates. Brain Res Bull 68: 185–194. 16325019 10.1016/j.brainresbull.2005.08.010

[79] DeMonasterio FM, Schein SJ, McCrane EP (1981) Staining of blue-sensitive cones of the macaque retina by a fluorescent dye. Science 213: 1278–1281. 7268439 10.1126/science.7268439

[80] Martin PR, Grünert U (1999) Analysis of the short wavelength-sensitive (“blue”) cone mosaic in the primate retina: Comparison of New World and Old World monkeys. JCompNeurol 406: 1–14. 10.1002/(sici)1096-9861(19990329)406:1<1::aid-cne1>3.0.co;2-1 10100889

[81] Peichl L, González-Soriano J (1994) Morphological types of horizontal cell in rodent retinae: A comparison of rat, mouse, gerbil, and guinea pig. VisNeurosci 11: 501–517. 10.1017/s095252380000242x 8038125

[82] Li W, DeVries SH (2004) Separate blue and green cone networks in the mammalian retina. Nat Neurosci 7: 751–756. 15208635 10.1038/nn1275

[83] Kennedy MJ, Davison IG, Robinson CG, Ehlers MD (2010) Syntaxin-4 defines a domain for activity-dependent exocytosis in dendritic spines. Cell 141: 524–535. 20434989 10.1016/j.cell.2010.02.042 PMC2874581

[84] Haverkamp S, Grünert U, Wässle H (2001) Localization of kainate receptors at the cone pedicles of the primate retina. J Comp Neurol 436: 471–486. 11447590 10.1002/cne.1081

[85] Haverkamp S, Grünert U, Wässle H (2001) The synaptic architecture of AMPA receptors at the cone pedicle of the primate retina. J Neurosci 21: 2488–2500. 11264323 10.1523/JNEUROSCI.21-07-02488.2001 PMC6762391

[86] Puller C, Haverkamp S, Grünert U (2007) OFF midget bipolar cells in the retina of the marmoset, Callithrix jacchus, express AMPA receptors. J Comp Neurol 502: 442–454. 17366611 10.1002/cne.21315

[87] Herr S, Klug K, Sterling P, Schein S (2003) Inner S-cone bipolar cells provide all of the central elements for S cones in macaque retina. J Comp Neurol 457: 185–201. 12541318 10.1002/cne.10553

[88] Kouyama N, Marshak DW (1992) Bipolar cells specific for blue cones in the macaque retina. JNeurosci 12: 1233–1252. 1556594 10.1523/JNEUROSCI.12-04-01233.1992 PMC6575807

[89] Calkins DJ, Tsukamoto Y, Sterling P (1998) Microcircuitry and mosaic of a blue-yellow ganglion cell in the primate retina. JNeurosci 18: 3373–3385. 9547245 10.1523/JNEUROSCI.18-09-03373.1998 PMC6792640

[90] Dacey DM, Lee BB (1994) The ‘blue-on’ opponent pathway in primate retina originates from a distinct bistratified ganglion cell type. Nature 367: 731–735. 8107868 10.1038/367731a0

[91] Ghosh KK, Grünert U (1999) Synaptic input to small bistratified (blue-ON) ganglion cells in the retina of a new world monkey, the marmoset Callithrix jacchus. J Comp Neurol 413: 417–428. 10502249 10.1002/(sici)1096-9861(19991025)413:3<417::aid-cne5>3.0.co;2-h

[92] Percival KA, Jusuf PR, Martin PR, Grünert U (2009) Synaptic inputs onto small bistratified (blue-ON/yellow-OFF) ganglion cells in marmoset retina. J Comp Neurol 517: 655–669. 19830807 10.1002/cne.22183

[93] Crook JD, Davenport CM, Peterson BB, Packer OS, Detwiler PB, et al (2009) Parallel ON and OFF cone bipolar inputs establish spatially coextensive receptive field structure of blue-yellow ganglion cells in primate retina. J Neurosci 29: 8372–8387. 19571128 10.1523/JNEUROSCI.1218-09.2009 PMC2733872

[94] Field GD, Sher A, Gauthier JL, Greschner M, Shlens J, et al (2007) Spatial properties and functional organization of small bistratified ganglion cells in primate retina. J Neurosci 27: 13261–13272. 18045920 10.1523/JNEUROSCI.3437-07.2007 PMC6673390

[95] Dacey DM (1999) Primate retina: cell types, circuits and color opponency. Prog Retin Eye Res 18: 737–763. 10530750 10.1016/s1350-9462(98)00013-5

[96] Dacey DM (1993) Morphology of a small-field bistratified ganglion cell type in the macaque and human retina. Vis Neurosci 10: 1081–1098. 8257665 10.1017/s0952523800010191

[97] McMahon MJ, Packer OS, Dacey DM (2004) The classical receptive field surround of primate parasol ganglion cells is mediated primarily by a non-GABAergic pathway. J Neurosci 24: 3736–3745. 15084653 10.1523/JNEUROSCI.5252-03.2004 PMC6729348

[98] Schwartz EA (1987) Depolarization without calcium can release y-aminobutyric acid from a retinal neuron. Science 238: 350–355. 2443977 10.1126/science.2443977

[99] Pow DV, Baldridge W, Crook DK (1996) Activity-dependent transport of GABA analogues into specific cell types demonstrated at high resolution using a novel immunocytochemical strategy. Neuroscience 73: 1129–1143. 8809830 10.1016/0306-4522(96)00097-8

